# Supplementary material for: Learning curve and surgical outcome of robotic assisted colorectal surgery with ERAS program
Source: Sci Rep. 2022 Nov 29;12:20566. doi: 10.1038/s41598-022-24665-w (PMC9709162; doi:10.1038/s41598-022-24665-w)
Supplement: Supplementary file 1 — Supplementary Information 1. [file 41598_2022_24665_MOESM1_ESM.zip › LSC Raw data-Ñ[▒K/056/HS_ñw╝╨░O▒Kñσ.pdf]

正式摘要(電子簽章：[illegible])

W55 / W55 ☐ 英文標題(注意:無英文姓名-請補病人基本檔!) ☐【讀取】憑證

臺中榮民總醫院

出院病歷摘要

[redacted]

(8)流水編號	
(9)入院日期	108/12/11 (2019) 直腸外科 W55 - 265
(10)轉科日期	
(11)出院日期	108/12/19 (2019) 住院天數計 8 日

診斷

- 入院 :Rectal cancer, s/p CCRT
- 出院 :Rectal cancer, s/p CCRT, s/p Laparscopic Total mesocolon excision on 20191212, ycT3N0M0

主訴

- Difficult in defecate for 1 week

病史

- This 61 yeard old male patient had history of
  1. Rectal cancer s/p neoadjuvant CCRT on 2019/10/24-30
  2. CAD-I s/p stent*1 in 2018/08
  3. Urethral stone s/p ESWL and scopic removal
  4. DM
  5. HTN

  According to the patient, he had difficult in defecate for weeks, so he went to LMD for help. He denied fever, nausea or vomiting, bloody stool. He was diagnosed as Rectal cancer and transfer to our hospital for further treatment. Then, he received neoadjuvant CCRT in our hospital.
  This time, he was admitted for CT follow up and arranged surgery.

體檢發現

- Anal inspection:
  Recto digital examination: L/3
  - General appearance :
    - easy looking , conscious clear , without cardiopulmonary distress
    - Vital signs自動帶入date 體溫(BT) 脈搏(P) 呼吸(R) 血壓(BP) 身高(BH) 體重(BW) BMI
      20191211 35.5℃ 84次/分 18次/分 160/94mmHg 174cm 74.7kg 24.7
    - Vital signs: B.P. 160 mmHg , P.R. 84 /min , R.R. 18 /min , B.T. 35.5 ℃
  - HEENT :
    - Eyes: no anemic conjunctiva, no icteric sclera, normal EOM
    - ENT: no otorrhea, no rhinorrhea, no erythematous mucosa, no injected tonsil, no deformity
  - Neck: supple, freely movable, trachea midline,no deformity, no engorged jugular vein , no palpable thyroid or lymph node enlargement
  - Chest:
    - Inspection: Grossly normal, symmetric and fully expansion, no accessory muscle used, no operation scar, no spider angiomata, no gynecomastia
    - Palpation: no palpable mass or nodule, no subcutaneous emphysema, no tenderness
    - Ausculation: clear breathing sound
  - Heart:
    - Palpation: no thrill, no heave
    - Percussion: normal heart size
    - Ausculation: regular heart beat, normal S1, S2, no gallop, no murmur
  - Extremities: No deformity, full ranged of motion without limitation, no clubbing finger or nail cyanosis, no pitting edema, no ulcer or pigmentation, no obvious skin lesion, no varicose vein, no bone, joint or muscle tenderness

CRS CONDITION

- Karnofsky performance status: (100) Normal, no complaints, no evidence of disease
- PE of Abdomen:
- CEA: 6.65 ng/ml
- Imaging Studies:
- Colonoscope report: .
- Figures:
  1. colon & rectum

手術日期及方法

- 手術日期及方法20191212 1. Laparoscopic assisted low anterior resection with end-to-end anastomosis, EndoGIA 60/4.1mm*2, CDH 33mm, re-inforcement of silk (-), air leak test : no leakage.
  2. High ligation IMA below left colic artery.
  3. Cut margin: proximal 15 cm, distal 1.5 cm.
  4. Take down of splenic flexure: (-)
  5. A Fr.12 Penrose drain placed in the pelvis.
  6. Loop ileostomy on RLQ.
  20191212 Ureter, Double J insertion
- 手術結果及發現 :
  - 1. One 0.5x0.5cm Borrmann type II tumor at lowe rectum, which invaded through the muscular propria and into pericolorectal tissue. no enlarged lymph nodes at mesorectum were noted, ycT3N0M0. 2. There was no palpable liver metastasis, no visible peritoneal seeding.

住院治療經過

- <12/11~12/17> After admission he received Laparoscopic low anterior resection on 2019/12/12. We remove NG on 12/13. And we let him try water on 12/14, gave him PPI. Perdipine pump was used due to hypertension, and we let him try bland I diet on 12/16. We let him try bland II diet on 12/17. <12/18~12/19> We let him try bland III diet on 12/18 and remove the penrose drain. Due to stable condition, we let him dicahrge on 2019/12/19 and arrange OPD follow up.

合併症

一般檢查

- 累積報告 :

DATE	TIME	ALB	TP	BIL.T	BIL.D	ALKP	AST	ALT	LDH	CHOL	TG	HDL-C	LDL	UA	NH3	AMY
1081211	1817						20	17								
1081216	0915						13	10								

DATE	TIME	WBC	RBC	HGB	HCT	MCV	PLT	BLST	NEUT	BAND	SEG	LYM	MON	EOS	BAS
1081211	1817	6200	4.67	14.3	42.3	90.6	147		59.9			29.5	7.9	2.4	0.3
1081216	0927	6230	4.86	14.5	43.8	90.1	147		69.1			18.3	9.0	3.0	0.6

DATE	TIME	NA	K	CL	CA	BUN	CREAT	Mg	P	CRP	ESR	PCT	LIPASE	LACTATE
1081211	1817	136	3.9			21	1.32							
1081216	0915	134	4.5			22	1.19							

DATE	TIME	GLUCOSE	PAB	TRF	ZN	CU	KETONE	CK	CKMB	PT-P	PT-C	APTT-P	APTT-C	TRO-T
1081211	1816									10.6	10.1	31.0		
1081216	0939									10.8	10.1	30.5		

- 放射線報告 :檢驗項目 **:CT, PELVIS** 報告日期 **:1081211**
  主 訴 :

- Result:
 Finding:
 Imaging Report Form for Colorectal Carcinoma (AJCC 8th)
 1. Imaging Date: 20191211
 Imaging Modality, Imaging by
 CT scan
 Imaging Protocol:
 Post-contrast CT scan of abdomen with IV contrast administration and oral contrast ingestion
 2. Tumor location / Size
 Location:
 Rectum
 Size:
 Non-measurable
 3. Tumor invasion
 Tumor cannot be visualized in this imaging study
 4. Regional nodal metastasis
 No
 5. Distant metastasis (In this study)
 No or Equivocal
 6. Other findings:
 >The primary tumor of rectum reveals regressive change as compared with simulation CT on 2019/10/23.
 >Two stones(0.7-0.9cm,Ser203-img28) in left upper-third ureter are mentioned with mild left hydronephroureter and increased left perinephric strandings. Left pyelonephritis can not be ruled out.
 >A cyst(length-1.5cm) in upper pole of right kidney
 >Mild enlargement of prostate gland with internal small calcifications
 >Atherosclerotic calcified plaque formation of abdominal aorta, left renal artery orifice and bilateral common iliac arteries
 >DJD change with marginal spur formation of L-spine
 Impression:
 >Rectal cancer reveals regressive change as compared with simulation CT on 2019/10/23.
 >Left upper-third ureteral stones with mild left hydronephroureter and left pyelonephritis

 檢驗項目 **:CHEST PA VIEW** 報告日期 **:1081211**
 主 訴：
 Result:
 Finding:
 1. The heart size is within normal limit with tortuous aorta
 2. No obvious active lung lesion.
 3. minimal Pleural thickening over both lung apices
 4. mild DJD changes over T-L spine
 Impression:
 As above description.

特殊檢查

病理報告

- 活體組織切片病理報告 手術日期：**108/12/12** 病理號：**S0844727**

診斷醫師:吳致瑩 報告日期:108/12/19
Pathologic diagnosis:
1. Rectum, laparoscopic low anterior resection --- Status post neoadjuvant therapy. Adenocarcinoma, moderately differentiated. Tumor invaded to muscularis propria. The proximal cut end and perirectal lymph nodes (0/23) are free of tumor.
2. IMA root, dissection --- Unremarkable lymph nodes (0/6).
3. Rectum, cut end, distal, resection --- Mixed hemorrhoid. No tumor seen.
Ancillary study for diagnosis:
IHC stains for MLH1, MSH2, PMS2 and MSH6 are done on section E. All above markers are preserved.
Prognostic and predictive factor:
1. Tumor size: 0.7 cm (measure on slide).
2. Depth of invasion: Muscularis propria.
3. Histologic grade: G2: Moderately differentiated.
4. Angiolymphatic invasion: Not identified.
5. Perineural invasion: Not identified.
6. Tumor deposits (discontinuous extramural extension): Not identified.
7. Surgical Margins: a) Proximal Margin: Uninvolved by invasive carcinoma, distance of tumor from margin: 11.5 cm. b) Distal Margin: Uninvolved by invasive carcinoma, distance of tumor from margin: 0.5 cm. c) Radial or Mesenteric Margin: Uninvolved by invasive carcinoma, distance of tumor from margin: 3.5 cm.
8. Lymph node status: Uninvolved (0/29).
9. Extranodal involvement: Not applicable.
10. Tumor regression grading S/P CCRT: residual cancer with evident tumor regression, but more than single cells or rare small groups of cancer cells (partial response, score 2).
11. Type of polyp in which invasive carcinoma arose: Not applicable.
12. Additional pathologic findings: Mixed hemorrhoid.
13. Pathological TNM Stage: ypT2N0 (According to the eighth edition, 2017, American Joint Committee on Cancer Staging Guidelines for Tumors / 2017 CAP guideline).
14. TNM descriptors: y (Post-treatment).
Gross description:
The specimen consists of 1) a segment of rectum, 13 cm in length and 6 cm in proximal cut end circumference, 7.2 cm in distal circumference, and perirectal soft tissue. A faint and mildly ulcerative lesion, 1.5x1.2 cm, is noted, 0.5 cm to the distal cut end. On cross section, the tumor invades superficially with 35 mm to the radial margin, with no serosal retraction. The both cut ends are free of tumor grossly. 2) IMA root, a piece of tan yellow soft tissue, 4.8x2.5x1.8 cm. 3) distal cut end, a piece of tan mucosa-covered soft tissue, 2.8x2x2.5 cm. Representative part for section: A-B) specimen 2 C) specimen 3 D) proximal cut end E-H) tumor J-L) LN.
Note:
1. The interpretation of MMR panel and recommended studies are as below: (1) No loss of nuclear expression of MMR proteins: low probability of microsatellite instability-high (MSI-H) . (2) Loss of nuclear expression of MLH1 and PMS2: testing for methylation of the MLH1 promoter and/or mutation of BRAF is indicated (the presence of a BRAF V600E mutation and/or MLH1 methylation suggests that the tumor is sporadic and germline evaluation is probably not indicated; absence of both MLH1 methylation and of BRAF V600E mutation suggests the possibility of Lynch syndrome, and sequencing and/or large deletion/duplication testing of germline MLH1 may be indicated). (3) Loss of nuclear expression of MSH2 and MSH6: high probability of Lynch syndrome (sequencing and/or large deletion/duplication testing of germline MSH2 may be indicated, and, if negative, sequencing and/or large deletion/duplication testing of germline MSH6 may be indicated). (4) Loss of nuclear expression of MSH6 only: high

- probability of Lynch syndrome (sequencing and/or large deletion/duplication testing of germline MSH6 may be indicated). (5) Loss of nuclear expression of PMS2 only: high probability of Lynch syndrome (sequencing and/or large deletion/duplication testing of germline PMS2 may be indicated). (6) Please note: There are exceptions to the above IHC interpretations. These results should not be considered in isolation, and clinical correlation with genetic counseling is recommended to assess the need for germline testing.
  2. This case has been peer reviewed by two doctors.
  #T-59600_2 #M-81403_2 2 1105 M0SP0F
  #T-C4520_2 #M-09183_2 0 1105 000000

其它

出院時情況

- 3.改門診治療

出院指示

藥名	劑量	劑量單位	用法	頻次	數量	數量單位	現況	天數	單位
Panho tab 40mg	1	TAB	PO	QDAC	7	TAB	TH	7	天
Ultracet tab	1	TAB	PO	Q6HPRN	28	TAB	TH	7	天
OCAS Harnalidge 0.4 mg	1	TAB	PO	QDAC	21	TAB	TH	21	天

出院計劃

- 門診日期
  - date on 2020/01/07
- 衛教 :Post oP wound care
- Arrange OPD follow up

主治醫師_2062F 蔣鋒帆_______ / 住院醫師_2339C 蔡濬[illegible]_______

正式摘要(電子簽章：**000000183333102)**

W55 / W55

doctorRank:B
